# Inequitable access to neurosurgical care in the United States

**DOI:** 10.3389/fneur.2026.1850876

**Published:** 2026-06-18

**Authors:** Linda Liverani, Nina Dwumfour-Poku, Corinna C. Zygourakis, Silvia D. Vaca

**Affiliations:** 1 Department of Neurosurgery, Stanford Health Care, Palo Alto, CA, United States; 2 School of Medicine, UT Southwestern, Dallas, TX, United States

**Keywords:** gender diversity, geographic disparities, neurosurgery training, neurosurgical access, rural neurosurgery, social determinants of health, telemedicine, United States

## Abstract

Despite a national neurosurgeon density of 1.58 per 100,000 (exceeding the WHO minimum threshold of 1 per 100,000), equitable access to neurosurgical care remains uneven in the United States. Approximately 9.8% of the US population lacks adequate access to neurosurgical services. Rural and socioeconomically disadvantaged communities are most affected. This literature review focuses specifically on the intersection of neurosurgical access and the distribution, composition, and pipeline of the US neurosurgical workforce. We conducted a narrative review of peer-reviewed literature from 2015 to 2025 using PubMed, Scopus, and Web of Science. Search terms included “neurosurgical workforce,” “geographic access,” “gender equity,” “rural neurosurgery,” “telemedicine,” and “task sharing.” Eighty percent of US counties lack neurosurgeons, and only 2.3% of neurosurgeons practice in nonmetropolitan areas. Unemployment, low educational attainment, and poverty independently predict reduced neurosurgeon availability. Geographic access and workforce diversity overlap. States with the fewest training programs also show the lowest representation of women in neurosurgery. Concurrently, graduating medical students interested in neurosurgery who were female, Black/African-American, or Hispanic were significantly more likely to report intention to practice in underserved areas. Addressing geographic, socioeconomic, and gender disparities simultaneously is essential to ensuring timely access to neurosurgical care for all patients. Telemedicine networks, rural training tracks, and intersectional recruitment strategies offer scalable pathways toward a more equitable workforce.

## 1 Introduction

Neurosurgery is among the most demanding and geographically concentrated surgical specialties. The aggregate US neurosurgeon density of 1.58 per 100,000 population exceeds the WHO threshold of one per 100,000, suggesting national sufficiency (1, 2). Yet this access is unevenly distributed. At the “Hospital Referral Region” level as the geographic unit representing a tertiary care region in which most Medicare patients stay to receive neurosurgical procedures - *median* density drops to 1.47 per 100,000. The *mean* number of neurosurgeons is 2.22 per 100,000, with wide variation across states and systematic underservice in rural and socioeconomically disadvantaged regions (3). Approximately 9.8% of the US population lacks adequate geographic access to neurosurgical care – defined as neurosurgeon density of at least 0.5 per 100,00 - a figure that translates to more than 32 million Americans (4).

The consequences of inadequate access are clinically and economically significant. Delayed treatment for conditions such as traumatic brain injury, aneurysmal subarachnoid hemorrhage, and spinal cord compression is associated with worse outcomes and higher rates of disability (3, 5). Beyond clinical harm, the financial burden of traveling long distances for neurosurgical care falls disproportionately on low-income and minority populations, compounding geographic barriers to access care (4).

Inequities in access to neurosurgical care are likely multifactorial - necessitating the expansion of systems, processes, infrastructure, and workforce to address this need. This literature review focuses specifically on the intersection of neurosurgical access and the distribution, composition, and pipeline of the US neurosurgical workforce. Geographic access and workforce diversity overlap. Adachi et al. (6) found that the number of residency programs in a state was a significant predictor of the number of female neurosurgeons practicing there. El Naamani et al. (7) showed that the ratio of women to total neurosurgery residents nearly doubled across 114 US programs, from 15.9% in 2016 to 29.8% in 2022, although representation varied widely by state and program. Racial and ethnic minorities also remain underrepresented across the specialty, with no significant improvement in Black or Hispanic faculty representation over the past decade (8).

Although the overall number of neurosurgeons has grown, this growth has not kept pace with population increases and has not been evenly distributed, leaving large portions of the country underserved (9). Concurrently, the neurosurgical workforce remains far from representative of the population. As of 2020, women represented only 9.6% of board-certified neurosurgeons in the United States (10), well below most other surgical specialties. The share of female residents grew from 15.1% in 2011 to 20.5% in 2020, but the workforce remains far from representative (8). Racial and ethnic minorities are similarly underrepresented: Hispanics, Black/African Americans, and Native Americans/Alaska Natives accounted for only 4.3, 4.5, and 0.2% of residents in 2019, respectively (11). These workforce characteristics may influence disparities in access to neurosurgical care.

These issues of geographic maldistribution and workforce diversity are interconnected. This literature review examines the current state of inequitable access to neurosurgical care in the United States through the lens of workforce composition, distribution, and training pathway across four domains: geographic and socioeconomic disparities in surgeon distribution, structural barriers perpetuating these disparities, the intersection of access and diversity, and evidence-based strategies for building a more equitable neurosurgical workforce. Although the United States is a high-income country, the magnitude of its internal disparities mirrors the global neurosurgical access deficit, making the US experience relevant to equitable health system reform worldwide (8, 9).

## 2 Methods

A narrative review of peer-reviewed literature was conducted using PubMed, Scopus, and Web of Science. Search terms included “neurosurgical workforce,” “neurosurgeon density,” “geographic access,” “rural neurosurgery,” “social determinants of health”, “gender equity in neurosurgery”, “telemedicine”, and “task sharing.” Studies published between January 2015 and December 2025 were included to reflect most recent developments in workforce trends. A total of 29 studies were included based on their relevance to the four thematic areas examined (Table 1).

**Table 1:** Summary of included studies.

Study details	Study design	Key findings
Vaca et al. (1)	Systematic review	Africa had the highest percentage of women neurosurgeons though fewer total neurosurgeons compared to other study populations. Middle- and high-income countries had a higher number of neurosurgeons compared to low-income countries through a marginal difference in the percent of women neurosurgeons compared to low-income countries
Mukhopadhyay et al. (2)	Mixed-methods	The global number of neurosurgeons is 49,940 with total density increased over the past decade
Rahman et al. (3)	Observational study	Regions with lower socioeconomic status in the U. S. have a lower density of neurosurgeons. Unemployment, low income and decreased rate of college graduates are also associated with lower density of neurosurgeons.
Perera et al. (4)	Cross-sectional study	Hispanic and Native Americans on average have a higher distance to neurosurgical care. Higher proportions of Hispanic residents is correlated with lower neurosurgeons per capita
Bhimani et al. (5)	Observational study (simulation-based)	Authors project neurosurgical workface shortages through the year 2037 with slightly higher adequacy in metropolitan areas compared to non-metropolitan areas.
Adachi et al. (6)	Retrospective observational study	Urban states have a higher percentage of women neurosurgeons compared to non-urban states. Additionally states with more training programs, female physicians and paid parental leave have a higher proportion of female neurosurgeons.
El Naamani et al. (7)	Cross-sectional study	The number of women matching into neurosurgery residency has increased modestly from 2016 (15.9%) to 2022 (29.8%), especially for international medical graduates.
Allison et al. (8)	Retrospective observational study	From the years 2012–2021 there has been no significant increase in the number of neurosurgery faculty coming from underrepresented groups, namely in race and gender.
Singh et al. (9)	Observational study	From 2017 to 2019, in all regions except for the West, the neurosurgeon per capita ratio (NCPR) has decreased.
Malueg et al. (10)	Cross-sectional study	Study revealed a positive correlation between percentages of female faculty and female trainees.
Maqsood et al. (11)	Observational study	Study finds ongoing gender and racial underrepresentation in neurosurgery training programs in the U. S. from 2007–2019
Meara et al. (43)	Report/policy paper	There exists a profound unmet surgical need in LMICs with surgical conditions causing increased morbidity and mortality.
Farivar et al. (12)	Observational study	Patients residing in counties with greater RUC codes and higher percentages of American Indian and Hispanic residents travel greater distances to access pediatric neurosurgeons.
Sarpong et al. (13)	Survey-based study	There is a statistically significant difference in the availability of subspecialty training in LMICs vs. HICs.
Kim et al. (14)	Narrative review	Paper argues that diversity is necessary for the advancement of neurosurgery and efforts must be taken to make the field more inclusive.
Naik et al. (29)	Cross-sectional study	Geographic hotspots of women neurosurgeons (WN) are a predictor of additional neurosurgeons entering the area.
Charles et al. (30)	Descriptive program evaluation study	Following the, “Future Leaders in Neurosurgery Symposium for Underrepresented Students” (FLNSUS) applicants showed statistically significant increased familiarity with the field, increased confidence in their abilities to become neurosurgeons, and increased exposure to neurosurgeons from diverse gender, racial, and ethnic backgrounds
Yoon et al. (31)	Prospective cohort study	For 310 patients receiving neurosurgery telemedicine services, mean overall satisfaction rate was 6.32 ± 1.27.
Yoon et al. (32)	Interventional study/quality improvement	Authors proposed a remote spinal examination that allows spine care providers to perform a nearly comprehensive exam through telemedicine.
Mukumbya et al. (33)	Interview-based study of Ugandan neurosurgeons and neurologists	Based on interview data, findings support the feasibility, appropriateness, and usability of mobile neuro clinics in providing care to remote Ugandan populations.
Morrison et al. (35)	Clinical case series, feasibility study	All endovascular robotic surgeries were successful (no conversion to manual) with 4 out of 6 aneurysms completely obliterated at 1-year follow-up, suggesting robotic neuroendovascular surgery is feasible and safe.
Olson et al. (36)	Feasibility study	Telerobotic-assisted coil embolization and thrombectomy with magnetic navigation was successful, suggesting that these procedures could be performed at long distances.
Hansen et al. (37)	Workshop with stakeholder discussions	Researchers developed structured research agenda following conclusion of discussions and concluded that unanswered questions remain on how telerobotic surgery can decrease the urban–rural healthcare gap.
Barrie et al. (39)	Survey-based study	“Neurosurgery cohort had fewer students identify as female, and report an intention to work with underserved populations. Black/African-American students were significantly more like to indicate intention to pursue Neurosurgery compared to White students. Black/African-American, Hispanic and female students were more likely to report an intention to practice in underserved urban and rural areas, compared to their peers.”
Mulligan et al. (40)	Retrospective registry analysis	Female neurosurgeons will not reach the proportion of women in the general medical workforce until 2,177.
Pugazenthi et al. (41)	Mixed-methods cross-sectional survey	Female medical students were more hesitant to pursue neurosurgery based on perceived maternal needs and technical skill required though were more influenced by shadowing experiences and elective rotations compared to their male counterparts.
Pugazenthi et al. (42)	Multi-institutional cross-sectional survey	Female medical students faced gender-specific barriers to pursuing neurosurgery (including maternity needs, finding identity-concordant mentors) while URM students faced race-specific barriers (not seeing people like them) that increased their expressed hesitancy to pursue neurosurgical careers. When grouped by both gender and race these effects were compounded.
Dewan et al. (44)	Mixed-methods epidemiological study	22.6 million people worldwide suffer from neurosurgical conditions with 23,000 additional neurosurgeons needed to address unmet cases in LMICs.
Park et al. (45)	Perspective/editorial article	Neurosurgical demand exceeds supply in LMICs with limited access due to decreased availability and affordability. Lack of access to basic surgical care causes 3 times as many deaths as HIV/AIDs, tuberculosis and malaria combined

### 2.1 Geographic and socioeconomic disparities in neurosurgeon distribution

The national density of 1.58 neurosurgeons per 100,000 US population masks substantial subnational variation (2, 3). Analysis at the Hospital Referral Region level reveals a median density of 1.47 per 100,000, with regions characterized by elevated poverty rates, high unemployment, and lower educational attainment consistently demonstrating lower neurosurgeon availability per capita (3).

The rural–urban divide is pronounced. Only 2.3% of the neurosurgical workforce practices in nonmetropolitan areas (5). Eighty percent of US counties lack neurosurgeons (4). Patients in these counties must travel to urban tertiary centers for time-sensitive procedures, and mortality increases with distance from the nearest neurosurgeon (4). Barriers to rural practice include inadequate hospital infrastructure, limited support staff, restricted continuing education, and professional isolation (5).

Pediatric neurosurgical access poses additional concerns. Large portions of the rural South and Mountain West lack access to pediatric neurosurgeons within acceptable travel distances, defined as an average of 39.7 miles within a surgeon cluster compared to an average of 189.2 miles in a surgeon desert (12).

Socioeconomic indicators compound geographic barriers. Perera et al. (4) demonstrated that countries with lower median household income, higher rates of lacking insurance, and lower educational attainment were significantly less likely to have practicing neurosurgeons, even after adjusting for population size. At the regional level, lower socioeconomic status as measured by poverty, unemployment, and rates of uninsurance is associated with fewer neurosurgeons per capita, showing that unequal access follows multiple forms of disadvantage (3).

### 2.2 Structural barriers perpetuating workforce inequities

#### 2.2.1 Training pipeline and residency distribution

Training programs concentrate in metropolitan academic centers, where 97.7% of the neurosurgical workforce is based (5). States with more residency programs have more practicing neurosurgeons (6), and programs with higher proportions of female faculty enroll more female residents (10). Singh et al. (9) documented a declining neurosurgeon-to-population ratio from 2017 to 2019 in the Northeast, Midwest, and South, suggesting a decreasing influx of newly trained surgeons to these areas.

Sarpong et al. (13) surveyed neurosurgical trainees across 69 countries and identified limited case volume, inadequate mentorship (rated 4.8/10), financial constraints, and geographic isolation as major barriers to training. In the US, neurosurgery residency was extended to a mandated 7 years in 2014 (9), and nearly all training occurs in metropolitan academic centers where 97.7% of the neurosurgical workforce is concentrated (5). This leaves few graduates with direct experience in nonmetropolitan practice.

#### 2.2.2 Compensation, liability, and institutional infrastructure

Rural practice faces additional challenges. Only 31% of non-metropolitan areas had an adequate workforce in 2022, projected to decline to 25% by 2037 (5). Limited professional opportunities, isolation, and inadequate infrastructure contribute to difficulty recruiting and retaining neurosurgeons in these settings (5). Trauma centers in multiple states have closed due to neurosurgeon shortages, and many hospitals in the Midwest and South have lost accreditation for the same reason (9).

#### 2.2.3 Gender-based discrimination and institutional culture

Structural barriers to gender equity in neurosurgery are well documented. Maqsood et al. (11) described a tripartite pattern of systemic obstruction in which women and racial minorities face a sticky floor that prevents entry into competitive roles, a broken ladder that interrupts career advancement at mid-level, and a glass ceiling that limits access to leadership and academic appointments. Longitudinal analysis of ACGME residency enrollment data confirms that gender and racial disparities persist: female residents increased from 10.6% in 2007 to 19.3% in 2019, yet representation of racial and ethnic minorities has not significantly improved over the same decade, with only 5.8% Hispanic, 4.8% Black/African American, and 0.13% Native American/Alaskan residents in 2019 (11).

Program-level factors modulate these experiences. Training programs with higher proportions of female faculty demonstrated significantly higher female resident enrollment and retention, consistent with a role model effect (10).

Compensation disparities add a financial dimension to gender inequity. Women earn less than male counterparts at equivalent career stages in equivalent specialties across all of academic medicine (14). However, this complex issue requires further evaluation within neurosurgery, as many factors may be at play ranging from academic promotion, referrals, and differences by subspecialty, compensation for differing types of academic work, and balancing responsibilities outside of work.

#### 2.2.4 Systems and policies influencing inequitable care access and gender-based workforce disparities

Inequitable access to neurosurgical care in the United States is multifactorial, influenced by insurance policy and socioeconomic factors that intersect with disparities in workforce distribution. Neurosurgical care access has been expressly influenced through narrow network plans, a byproduct of the Affordable Care Act (ACA) (15–17). Multiple studies have elucidated the state-level effects of these narrow networks on neurosurgical care. Jumah et al. (15) found that as of 2019 in the state of New Jersey, 25% of neurosurgeons did not participate in ACA plans. Similarly, in *Access to Neurosurgery in the Era of Narrowing Insurance Networks*, from 2016 to 2019, 10 of 15 counties in the state of Arizona did not have access to outpatient neurosurgical care through existing ACA plans (16). Similar deficiencies were found in the state of Louisiana (17). Principally, these data suggest system-wide discrepancies in access based on current healthcare policy which place medically vulnerable populations at the highest risk for receiving delayed or completely inaccessible neurosurgical care. Additional research is warranted to determine the individual, state, and nationwide effects of these healthcare policies in more recent contexts.

Medicaid has served as a historical safety net for medically vulnerable populations, though also presents limitations regarding provider access (18, 19). Patients with Medicaid have less access to neurosurgical care, present later in their disease course, and experience higher rates of postoperative complications for the treatment of neurosurgical conditions (20–22). Medicaid reimbursement, which is largely determined at the state level, has lower overall rates compared to both that of Medicare and private insurance which decreases incentives for provider enrollment (18). States with lower Medicaid reimbursement also have lower neurosurgery provider enrollment compared to states with higher Medicaid reimbursement (19).

Alternatively, Medicaid expansion, a central component of the ACA has been shown to be favorably associated with increased access to surgical care, including neurosurgical care. Lohrer et al. (23) compared patient-level outcomes in expansion and non-expansion states and found Medicaid expansion to be associated with a 7.5% increase in insurance coverage at the time of admission for common surgical conditions, obtaining earlier care in disease care and more optimal care. Similar outcomes have been shown with increasing access to elective spinal surgery (24).

#### 2.2.5 Policy through an ethics and justice framework

More recent developments in healthcare policy, namely the introduction of the One Big Beautiful Bill Act (OBBA) enacted July 4, 2025 have sparked debate into how the landscape of American healthcare will change in regard to accessing timely and adequate care. Some healthcare pundits argue that the changes to Medicaid and ACA marketplace coverage enforced by the bill, including increased Medicaid work requirements and restrictions on states to use provider taxes as a mechanism of funding will further decrease access by making it difficult to enroll and maintain eligibility for Medicaid. Lobbying efforts have been made by neurosurgery organizations such as the Congress of Neurological Surgeons (CNS) and American Association of Neurological Surgeons (AANS) against the OBBA for more balanced Medicaid reform (25, 26). Studies have posited how these policy changes will affect rural care collectively, though it is unclear how these policies will specifically impact neurosurgical care in all settings long-term (27). Personalized accounts and responses to the policy have been mixed within the neurosurgical and medical community at large (28).

## 3 Gender composition and access: intersectional disparities

The geographic distribution of female neurosurgeons is not uniform (6). Women neurosurgeons worldwide, and in the United States specifically, are disproportionately concentrated in states hosting high-ranked academic medical centers and large residency programs (6). This creates a paradox: states with the strongest neurosurgical infrastructure have higher female representation, yet these are already well-served urban settings. States with the greatest access deficits and fewest training programs show the lowest female representation (29). Naik et al. (29) found that counties classified as hot spots for gender diversity had a median of 22 neurosurgeons per county of which 3.21 of them were women while cold spots had a median of only 3 neurosurgeons with zero women, and areas without female neurosurgeons in 2015 did not gain any over the following 7 years.

This overlap between access gaps and gender underrepresentation reflects shared structural barriers. Inadequate infrastructure, professional isolation, and limited subspecialty support discourage practice in underserved settings. Women face additional barriers: safety concerns, childcare limitations, spousal career opportunities, and institutional climate (7).

El Naamani et al. (7) conducted a cross-sectional demographic study of 114 US neurosurgery residency programs and documented a significant upward trend in female resident representation, rising from 15.9% in 2016 to 29.8% in 2022, with sustained growth among international medical graduates. From the years 2007–2019, Maqsood et al. (11) found an absolute change in racial distribution of neurosurgery residents with a + 1.5% increase for Hispanic residents, +0.3% for Black/African Americans, −0.12% Native Americans/Alaskans, and −1.6% others.

Intersectional analyses accounting for race, gender, and geography remain sparse but critical. Physicians from racial and ethnic minority groups face compounding barriers to neurosurgery entry, particularly from historically underrepresented communities (11). Virtual symposia and pipeline programs show early promise in broadening the applicant pool, though long-term outcome data remain limited (30).

## 4 Strategies to expand neurosurgical access in underserved regions

### 4.1 Telemedicine and telerobotic surgery

During the COVID-19 pandemic, many hospital systems saw an explosion of telehealth services for outpatient neurosurgical visits (31, 32), which have provided the added benefit of ease of access for those traveling long distances for clinic appointments or those with transportation challenges. Telemedicine has the potential to extend neurosurgical expertise to geographically isolated patients without specialist relocation.

Mobile neurosurgical clinics piloted in sub-Saharan Africa have demonstrated feasibility and community acceptance for delivering consultative and procedural services to populations outside fixed facility catchment areas (33). Similar models could be adapted for rural US communities to reduce geographic barriers to non-emergent consultation; however implementation must be considered in light of barriers including misdiagnosis, technological literacy and individual ability to navigate digital platforms (34).

Telerobotic surgery could further reduce the rural–urban health care gap by allowing urban surgeons to operate on patients in remote settings. Early studies have shown promise in robotic neuroendovascular surgeries, but further investigation is needed for this to be a feasible widespread option (35, 36). To provide telerobotic services for neurosurgical procedures, many challenges remain, including advancements in robotic technology to allow for fully remote surgical operators, logistics of troubleshooting issues or complications in the operating room, education and credentialing requirements, network latency, and economic viability (37). Additionally, more robust clinical evidence, including large-center RCTs, are needed to make telerobotic a more clinically acceptable practice as many current studies lack long-term prognostic data (38). As these challenges are addressed, telerobotic platforms may extend the operative reach of urban neurosurgeons to remote settings (37).

### 4.2 Rural training track development

Expanding rural training opportunities is a priority. Sarpong et al. (13) found that limited case volume, inadequate mentorship, financial constraints, and geographic isolation are major barriers to neurosurgical training globally, with mentorship availability rated only 4.8 out of 10 by trainees worldwide. In the US, neurosurgery residency was extended to 7 years in 2014 (9), and 97.7% of the workforce is concentrated in metropolitan areas (5), limiting rural exposure during training. Expanding training collaborations between academic centers in metropolitan areas with community centers in rural areas may provide a mutually beneficial experience for both trainee exposure and rural neurosurgeon support.

Barrie et al. (39) found that graduating medical students interested in neurosurgery who were female (aOR 2.44), Black/African-American (aOR 7.66), or Hispanic (aOR 4.50) were significantly more likely to report intention to practice in underserved areas. Targeted recruitment and support of these students could help address rural workforce gaps.

## 5 Discussion

This review identifies shared structural barriers underlying disparities in neurosurgical access and workforce diversity. Eighty percent of US counties lack neurosurgeons (4), and only 31% of rural communities have an adequate neurosurgical workforce (5) Gender parity in the practicing US neurosurgical workforce is projected to take more than 150 years (40).

National aggregate workforce sufficiency masks these disparities. The US density of 1.58 neurosurgeons per 100,000 population exceeds the WHO threshold (2), yet 9.8% of the population falls below the minimum standard for neurotrauma coverage (4). This reflects maldistribution predominantly impacting patients affected by poverty, unemployment, and uninsurance (3, 4). To provide equitable access to neurosurgical care, further work is warranted to establish and strengthen systems, infrastructure, and neurosurgeon support in these areas.

The relationship between access and gender equity warrants closer examination. Women now account for approximately 49% of US medical school graduates yet represent only 10.73% of active neurosurgeons (29). Female medical students considering neurosurgery are more hesitant than their male peers due to their belief in the existence of a glass ceiling within the profession, maternity concerns, role models and representation in the field, opportunities to pursue health equity work, and perceived technical skill requirements (41, 42). Women neurosurgeons concentrate in urban academic centers (1, 29), especially in the Middle Atlantic and Pacific divisions (29). Adachi et al. (6) found that the state diversity index was the only independent predictor of female neurosurgeon representation. The limited availability of childcare and mentorship in rural settings may further discourage women from practicing in underserved areas (41, 42). However, within the neurosurgery cohort, female students were 2.44 times more likely than male students to report intention to practice in underserved areas (39). Rural states also showed the highest percentage growth in female neurosurgeons between 2017 and 2023, with Kentucky (300%), Iowa (250%), and Arkansas (250%) leading (6). These trends suggest that addressing the glass ceiling, availability of childcare in rural settings and ensuring adequate and early mentorship given the intention of female students to practice in underserved areas may indirectly address disparities in access to neurosurgical care in metropolitan versus non-metropolitan areas within the United States, however; further studies are needed to evaluate whether gender representation meaningfully and independently decreases disparities in access to care. Similarly, studies suggest that URM medical students interested in neurosurgery, namely, Black/African and Hispanic students are more likely to express intent to practice in underserved and rural areas which suggests that addressing underrepresentation in neurosurgery training may likewise address disparities in neurosurgical care access (23). The US experience mirrors global patterns: although national neurosurgeon density exceeds WHO thresholds, regional gaps in access are similar to those seen in low and middle income countries (4, 32, 43).

## 6 Limitations

The narrative approach introduces selection bias, and the underlying literature is subject to publication bias toward large academic centers. Few studies in this literature employ longitudinal designs or report on long-term outcomes of workforce interventions, making it difficult to establish causal pathways from intervention to equity outcome. Future research should prioritize prospective evaluations of rural training tracks, telemedicine program effectiveness, and intersectional recruitment strategies.

## 7 Conclusion

Neurosurgical workforce inequity in the United States manifests as a convergence of geographic maldistribution, structural barriers to gender and racial diversity, and inadequate infrastructure in underserved settings. Eighty percent of US counties have no neurosurgeons, with rural communities carrying the greatest burden of unmet need. Gender parity in the specialty remains decades away at current rates. Addressing structural barriers in neurosurgical training and increasing gender diversity in the workforce present opportunities to make neurosurgical care more accessible across all regions and socioeconomic conditions.
